# *Lactobacillus* Ameliorates SD-Induced Stress Responses and Gut Dysbiosis by Increasing the Absorption of Gut-Derived GABA in Rhesus Monkeys

**DOI:** 10.3389/fimmu.2022.915393

**Published:** 2022-07-07

**Authors:** Ning Zhao, Yan Shu, Chenxing Jian, Zili Zhou, Haijun Bao, Xianguo Li, Xukai Cheng, Ying Zhao, Si Jin, Xiaogang Shu

**Affiliations:** ^1^Department of Gastrointestinal Surgery, Union Hospital, Tongji Medical College, Huazhong University of Science and Technology, Wuhan, China; ^2^Department of Endocrinology, Institute of Geriatric Medicine, Liyuan Hospital, Tongji Medical College, Huazhong University of Science and Technology, Wuhan, China

**Keywords:** sleep deprivation, stress responses, gut dysbiosis, GABA, *Lactobacillus*

## Abstract

Sleep deprivation (SD) has become a health problem in the modern society. Although probiotics supplementation has been proven to improve SD-induced gut dysbiosis, the potential neuroendocrine mechanisms remain elusive. In this study, thirty rhesus monkeys (RMs) were recruited. Paradoxical sleep, bright light, and noise were used to build an RM SD model. We examined the plasma γ-aminobutyric acid (GABA), stress hormones, and inflammatory cytokines using ELISAs. 16S ribosomal DNA sequencing and untargeted metabolomics sequencing were employed to detect gut microbial community and metabolites, respectively. The results of our study showed that RMs subjected to SD had elevated plasma stress hormones (such as cortisol and norepinephrine) and proinflammatory cytokines (such as TNF-α, IL-6, and IL-8), and a decreased anti‐inflammatory cytokine IL-10 level. Additionally, SD could give rise to a significant change in gut microbiota and metabolites. The differential gut microbiota and metabolites caused by SD were enriched in the signaling pathways related to GABA metabolism. Pearson correlation analysis revealed that there is a significant correlation between plasma GABA and SD-induced stress responses and gut dysbiosis. The supplementation of GABA-producing probiotics could significantly increase the relative abundance of *Lactobacillus* and plasma GABA levels, and reverse SD‐induced stress responses and gut dysbiosis. Therefore, we speculated that SD-induced stress response and gut dysbiosis might be an outcome of reduced gut-derived GABA absorption. The supplementation of GABA-producing *Lactobacillus* might be beneficial for the treatment of SD-induced intestinal dysfunction.

## Introduction

Sleep occupies approximately one-third of the human lifetime, and it plays a crucial role in maintaining physical and mental health (1). Nevertheless, nearly one-third of adults suffer from insufficient sleep due to social and family demands (2). As a strong stressor, sleep deprivation (SD) could lead to neuroendocrine dysfunction and metabolic disorders by activating the hypothalamic–pituitary–adrenal (HPA) axis (3) and the sympathetic–adrenal–medullary (SAM) system (4). Stress hormones such as cortisol and norepinephrine (NE) could exert downstream proinflammatory effects through stimulating macrophages and mast cells (5, 6). Gastrointestinal function is susceptible to endogenous and exogenous stressors (7). Some studies have confirmed that SD could cause excessive proinflammatory cytokines, gut microbiota disorders, and colitis phenotype in rats and *Drosophila* (8–10). However, the genetic identity, development traits, and physiological and psychological responses to stress in rats were far different from those in humans. Therefore, establishing rhesus monkey (RM) SD models was of great significance to study the consequences of SD in humans.

Gut symbiotic microorganisms are essential in the dynamic interaction between brain and gut (11, 12). Gut microbiota can affect numerous physiological pathways of the host, including nutrient metabolism, immune function, and the production of neurotransmitters as well as hormones (13). Probiotics is defined as live microorganisms that confer a health benefit on the host when ingested in adequate amounts. The most common beneficial effects of probiotics are restoring gut microbiota and improving intestinal and immune homeostasis (14, 15). Recently, some studies reported that probiotics could improve sleep quality and alleviate SD-induced memory and cognitive impairments (16, 17). In addition, *Lactobacillus*-based probiotics have also been reported to reduce the adverse effects of psychological stress (18). However, the neuroendocrine mechanisms by which probiotics supplementation improves SD-induced stress responses and gut dysbiosis remain elusive.

In this study, paradoxical sleep, bright light, and noise were used to build RM SD models. The level of plasma stress hormones, inflammatory cytokines, and gut microbiota and metabolites was used to assess the establishment of the RM SD model. The effect of probiotics supplementation on SD-induced stress responses and gut dysbiosis was then investigated.

## Materials and Methods

### Animals and Experimental Design

The animal experimentation protocols were approved by the Ethics Committee of Tongji Medical College of Huazhong University of Science and Technology, Wuhan, China (Approval No. S2536). All animal experiments were performed according to the Tongji Medical College Animal Care and Use Guidelines.

Thirty RMs (male, 6–7 years of age, 5.3–6.7 kg, from Hubei Tianqin Biotech Company) were randomized into a normal control group (NC group, *n* = 10) and an SD group (*n* = 20). They were housed in two different rooms more than 100 m away from each other. All RMs were kept under 12-h light/12-h dark conditions and a controlled temperature of 25–30°C and a controlled humidity of 50%–60%. Food and water are available *ad libitum*. All RMs were habituated to the housing condition for 5 days prior to the experiment. For control RMs, the living conditions remain unchanged. For SD RMs, bright light (12 h), noise (12 h), and paradoxical sleep were randomly combined to disrupt the sleep rhythm of RMs for 30 consecutive days.

Subsequently, fourteen RMs were randomly selected from twenty SD RMs and were randomly allocated into an SD control group (SD-NC group, *n* = 8) and a probiotics supplementation group (SD-Pro group, *n* = 6). All SD RMs were kept under 12-h light/12-h dark conditions. For control SD RMs, no additional interventions were added. For SD RMs in the probiotics supplementation group, Bifid Triple Viable Capsules (BTVC, 420 mg, bid) were added in the drinking water for 30 consecutive days. Bifid Triple Viable Capsules consisted of *Bifidobacterium*, *Lactobacillus*, and *Enterococcus faecalis*.

After the period of SD and probiotics intervention, the stool samples were collected and cryopreserved at −80°C. The blood samples were collected at 8:00 a.m. Subsequently, the plasma was extracted from the centrifuged blood samples (at 3,000 rpm at 4°C for 20 min) and then stored at −80°C.

### Enzyme-Linked Immunosorbent Assay

The detection of plasma γ-aminobutyric acid (GABA) (3302148206, Cloud-Clone), NE (1C05196326, Cloud-Clone), cortisol (CSB-EQ027342MK, CUSABIO), IL-6 (CSB-E10050Mo, CUSABIO), IL-8 (CSB-E08051Mo, CUSABIO), IL-10 (0BDA9BA014, Cloud-Clone), and TNF-α (CSB-E10039Mo, CUSABIO) was conducted *via* ELISAs.

### DNA Extraction and 16S Ribosomal DNA Gene Sequencing

The microbial community DNA was extracted using MagPure Stool DNA KF kit B (Magen China) following the manufacturer’s instructions. DNA was quantified with a Qubit Fluorometer by using the Qubit dsDNA BR Assay kit (Invitrogen, USA), and the quality was checked by running aliquot on 1% agarose gel. Variable regions V3–V4 of bacterial 16S rDNA were amplified with degenerate PCR primers, 341F (5’-ACTCCTACGGGAGGCAGCAG-3’) and 806R (5’-GGACTACHVGGGTWTCTAAT-3’). Both forward and reverse primers were tagged with Illumina adapter, pad, and linker sequences for 10 min. The PCR products were purified with AmpureXP beads and eluted in elution buffer. Libraries were qualified by the Agilent 2100 bioanalyzer (Agilent USA). The validated libraries were used for sequencing on the Illumina MiSeq platform following the standard pipelines of Illumina, and generating 2×300 bp paired-end reads. The Illumina MiSeq was made in USA and the 16S Ribosomal DNA Gene Sequencing was performed at BGI, China.

To obtain the effective tags, the raw tags of sequences were spliced and filtered. The effective tags were clustered into operational taxonomic units (OTUs) with >97% similarity using UPARSE (19). The α-diversity was assessed by ACE and Chao index using the vegan package. The β-diversity was assessed by calculating the weighted UniFrac distances matrix and Bray–Curtis distance matrix dissimilarity, which was visualized through the use of principal coordinate analysis. The differential taxa at the phylum, family, and genus levels were identified by linear discriminant analysis effect size (LEfSe) analysis. The Kyoto Encyclopedia of Genes and Genomes (KEGG) was used for the functional annotation of differential microbial community.

### Untargeted LC-MS-Based Metabolomic Analysis

Metabolite extraction was primarily performed according to previously reported methods (20). The samples were analyzed on a Waters 2D UPLC (Waters, USA), coupled to a Q-Exactive mass spectrometer (Thermo Fisher Scientific, USA). Chromatographic separation was performed on a Waters ACQUITY UPLC BEH C18 column (Waters, USA), and the column temperature was maintained at 45°C. The mobile phase consisted of 0.1% formic acid (A) and acetonitrile (B) in the positive mode, and in the negative mode, the mobile phase consisted of 10 mM ammonium formate (A) and acetonitrile (B). The gradient conditions were as follows: 0–1 min, 2% B; 1–9 min, 2%–98% B; 9–12 min, 98% B; 12–12.1 min, 98% B to 2% B; and 12.1–15 min, 2% B. The flow rate was 0.35 ml/min, and the injection volume was 5 μl. The collision energy for positive ions and negative ions was set at 3.8 kV and 3.2 kV, respectively. In order to provide more reliable experimental results during instrument testing, the samples are randomly ordered to reduce system errors. A quality control sample is interspersed for every 10 samples.

Data processing was performed using The Compound Discoverer 3.1 (Thermo Fisher Scientific, USA) software, which mainly included peak extraction, peak alignment, and compound identification. Using the SIMCA-P software (version 11.0; Umetrics), the multivariate raw data are dimensionally reduced by principal component analysis (PCA) and partial least square discriminant analysis (PLS-DA) to analyze the intergroup differences. The cross-validation ANOVA was used to validate the prediction power of the model, and the final overfitting of our data was assessed by permutation tests (*n* = 200). Variable importance in the projection (VIP) was calculated based on the PLS-DA and used to rank the importance of differential metabolites. All metabolites with a *p*-value < 0.05 and VIP > 1 were considered statistically significant. Metabolite pathway analysis was performed using the online software MetaboAnalyst 5.0 (http://www.metaboanalyst.ca/).

### Statistical Analysis

In this study, all experiments were repeated three times. The SPSS 23.0 (SPSS Inc., USA) and R (version 4.0.0) were used for statistical analysis. All variables were described by mean ± standard deviation, unless otherwise specified. The plasma indicators and metabolites were compared using *t*-tests. Spearman correlation analysis among plasma stress hormones, cytokines, and gut differential microbial community was performed using R software. All statistical tests were two-sided, with statistical significance set at 0.05.

## Results

### SD Induced Stress Responses and Gut Dysbiosis in RMs

**Figure 1:**
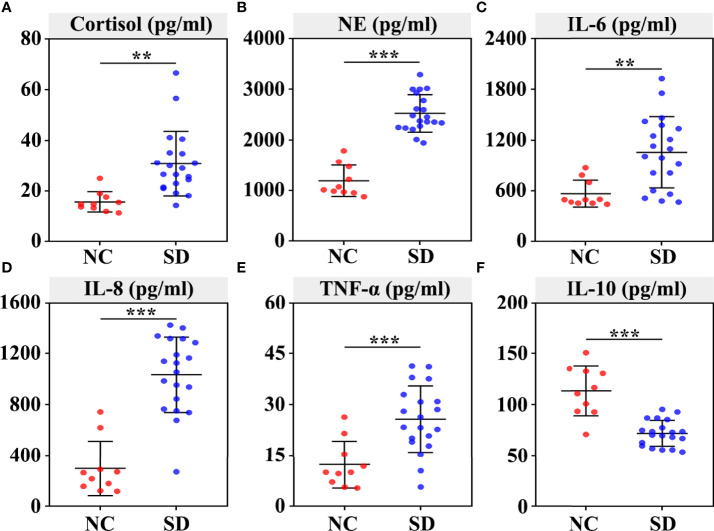
Effects of sleep deprivation (SD) on the secretion of stress hormones and inflammatory cytokines in rhesus monkeys. The plasma was extracted from the centrifuged blood samples after 30 days of sleep deprivation. The concentration of serum cortisol **(A)**, norepinephrine **(B)**, IL‐6 **(C)**, IL-8 **(D)**, TNF‐α **(E)**, and IL‐10 **(F)** was detected using ELISAs. Differences were denoted as follows: **p < 0.01; ***p < 0.001. NC, normal control group.

To explore whether SD induced stress responses and inflammatory reactions in RMs, we assessed the changes of plasma stress hormones and cytokines. We found that the level of plasma cortisol (*p* < 0.01) and NE (*p* < 0.001) was significantly increased in the SD group (**Figures 1A, B**). In addition, a marked increase of plasma proinflammatory cytokines (such as TNF-α, IL-6, and IL-8) and a reduction of plasma anti-inflammatory cytokine IL-10 was found in the SD group (**Figures 1C–F**).

**Figure 2:**
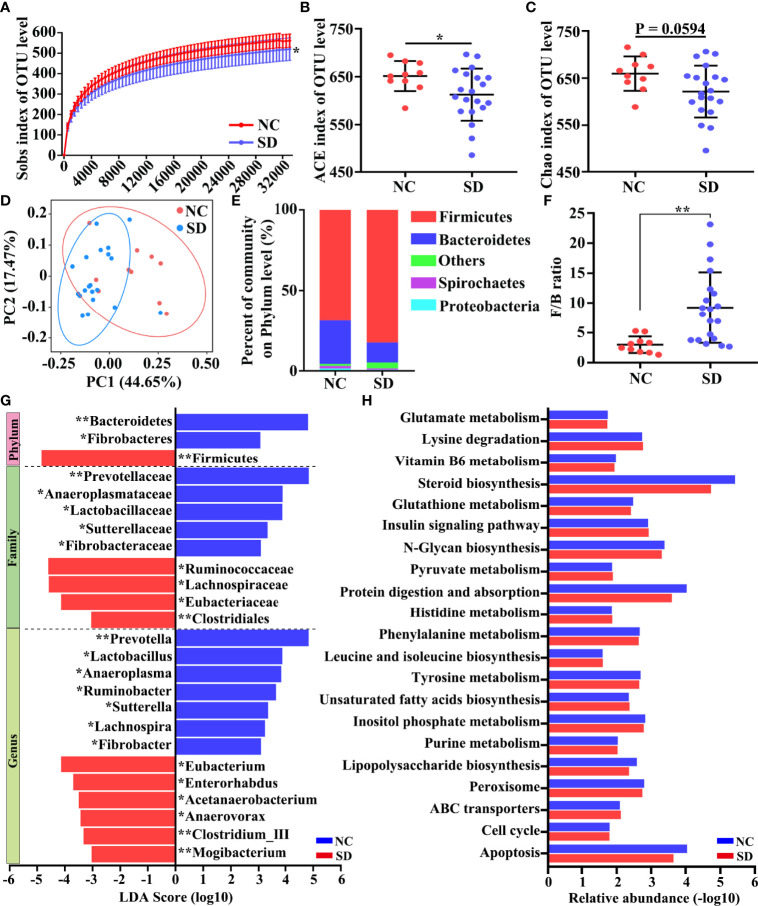
Effects of sleep deprivation (SD) on intestinal microbial community structure in rhesus monkeys (RMs). **(A)** Rarefaction curves for the gene number in the control (*n* = 10) and SD (*n* = 20) groups. The curve in each group is nearly smooth with a sufficient amount of sequencing data and a few new undetected genes. **(B, C)** ACE and Chao indices were used to estimate the α-diversity of the gut microbiota community. **(D)** Principal coordinate analysis showed that there is a distinct clustering of microbial community composition between SD and the control group. **(E)** The relative abundance of gut microbiota at the phylum level was clustered into different groups. This analysis included only phyla with relative abundances greater than 0.5%. All OTUs with low abundances were grouped as “others”. **(F)** The ratio of Firmicutes to Bacteroidetes in the SD RMs and controls. **(G)** The differential microbiota community between these two groups was identified by LEfSe methods. The enriched taxa of SD RMs were indicated with a negative LDA score (red), and the enriched taxa of controls were indicated with a positive LDA score (blue). Only taxa meeting a significant LDA threshold value of >3 are shown. **(H)** The differential KEGG pathways based on differential bacteria were shown. Differences were denoted as follows: *p < 0.05; **p < 0.01. NC, normal control group.

Next, we assessed whether SD could affect the composition of the gut microbiota community. A total of 2,048,445 high-quality sequences from 30 samples were used for the analysis. The data of 16S rDNA sequencing are shown in **Table S1**. As shown in the Venn diagram, most of the microbiota community (902/1139) were common between the SD group and the control group (**Figure S1A**). The rarefaction curve demonstrated that the gene richness of the SD group is significantly lower than that of the control group (**Figure 2A**). Similarly, α‐diversity analysis revealed that the richness and diversity of gut microbiota in the SD group is markedly less than that in the control group (ACE index and Chao index) (**Figures 2B, C**). In addition, a distinct clustering of microbial community composition between these two groups was found by β‐diversity analysis (**Figure 2D**). We also compared microbial community structures through performing taxon-dependent analysis. At the phylum level, SD led to a significant decrease of the abundance of Bacteroidetes, and a marked increase of the proportion of Firmicutes (**Figure 2E**) and the ratio of Firmicutes/Bacteroidetes (**Figure 2F**). An alteration in the composition of gut microbiota was also found at the family and genus levels (**Figures S1B, C**). To investigate the key flora affected by SD in RMs, we compared gut microbiota composition between these two groups using LEfSe methods. Based on *p* < 0.05 and LDA score > 3, a total of 25 differential microbial communities at the phylum (*n* = 3), family (*n* = 9), and genus (*n* = 13) levels were identified (**Figure 2G**). Among these bacteria, the relative abundance of *Lactobacillus* was significantly decreased in the SD group. The KEGG results showed that SD-induced gut microbiota disorders could affect many important biological processes, including glutamate metabolism, Vitamin B6 metabolism, and steroid biosynthesis (**Figure 2H**).

**Figure 3:**
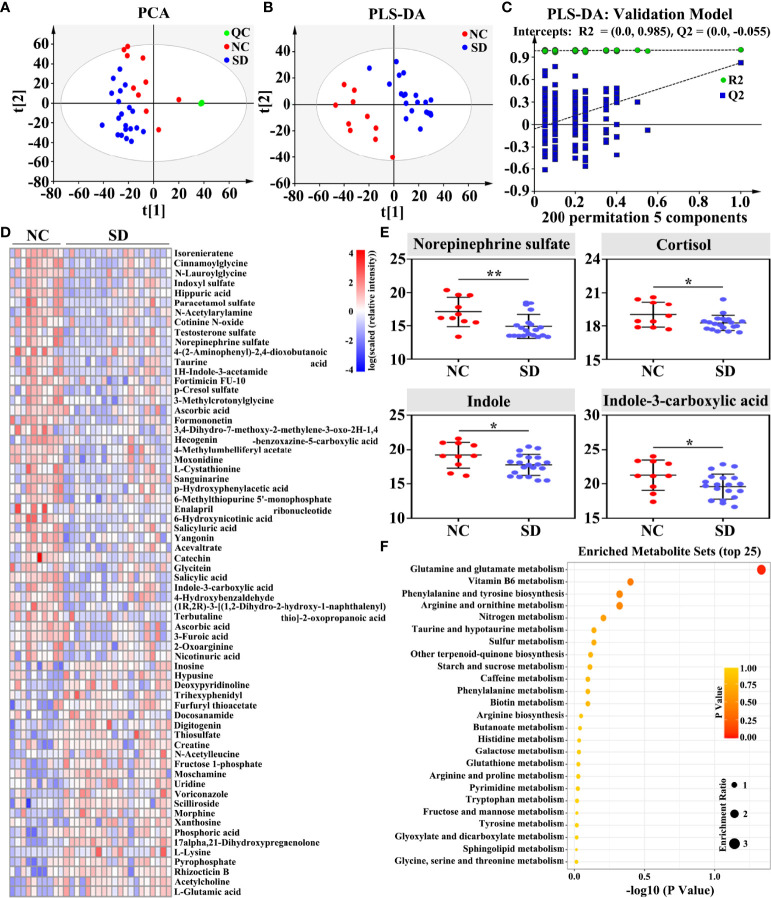
Effects of sleep deprivation (SD) on gut metabolite in rhesus monkeys (RMs). **(A)** The PCA score plot revealed that SD RMs can be clearly separated from controls by fecal metabolites (R2X = 0.674 and Q2 = 0.328). **(B)** The PLS-DA score plot demonstrated that fecal metabolites from SD RMs clustered well away those from controls (R2Y = 0.43 and Q2 = 0.825). **(C)** The results of the PLS-DA model were validated by the permutation test (*n* = 200, R2Y = 0.985, and Q2 = −0.055). **(D)** Sixty-six significantly differential metabolites (*p* < 0.05, VIP > 1 and |log(FC)| > 1.5) were shown in the heatmap. **(E)** Four metabolites implicated in stress response and tryptophan metabolism, namely, norepinephrine sulfate, cortisol, indole, and indole-3-carboxylic acid, were further compared and shown using a box map. **(F)** An overview of enriched metabolite sets for 227 differential metabolites using the online software MetaboAnalyst 5.0. Differences were denoted as follows: *p < 0.05; **p < 0.01. QC, quality control sample; NC, normal control group.

To explore the changes in metabolites induced by long-term SD, a metabolome program was carried out. A total of 4,143 metabolites were identified using the untargeted LC-MS method. As shown in the PCA plot, fecal metabolites can clearly distinguish SD RMs from non-SD RMs (**Figure 3A**). To exclude the influence of confounding variables, PLS-DA was further used to analyze gut metabolites. As shown in the PLS-DA plot, SD RMs were further separated from non-SD RMs (**Figure 3B**). The results of the PLS-DA model were validated by the permutation test (*n* = 200, R2Y = 0.985, and Q2 = −0.055) (**Figure 3C**). Finally, 227 differential metabolites (*p* < 0.05 and VIP > 1) were identified (**Table S2**) and 66 significantly changed metabolites with |log(FC)| > 1.5 were shown in the heatmap (**Figure 3D**). Among these differential metabolites, four metabolites related to stress hormones and tryptophan mechanism showed an obvious downward trend, including norepinephrine sulfate, cortisol, indole, and indole-3-carboxylic acid (**Figure 3E**). The KEGG results showed that glutamate metabolism, Vitamin B6 metabolism, and tryptophan mechanism were involved in SD-induced metabolic disorders (**Figure 3F**). Consistent with those findings in previous studies, our results supported that SD could lead to gut microbiota and metabolite disorders through activating the HPA axis and the SAM system.

### Plasma GABA Was Associated With SD-Induced Stress Responses and Gut Dysbiosis

**Figure 4:**
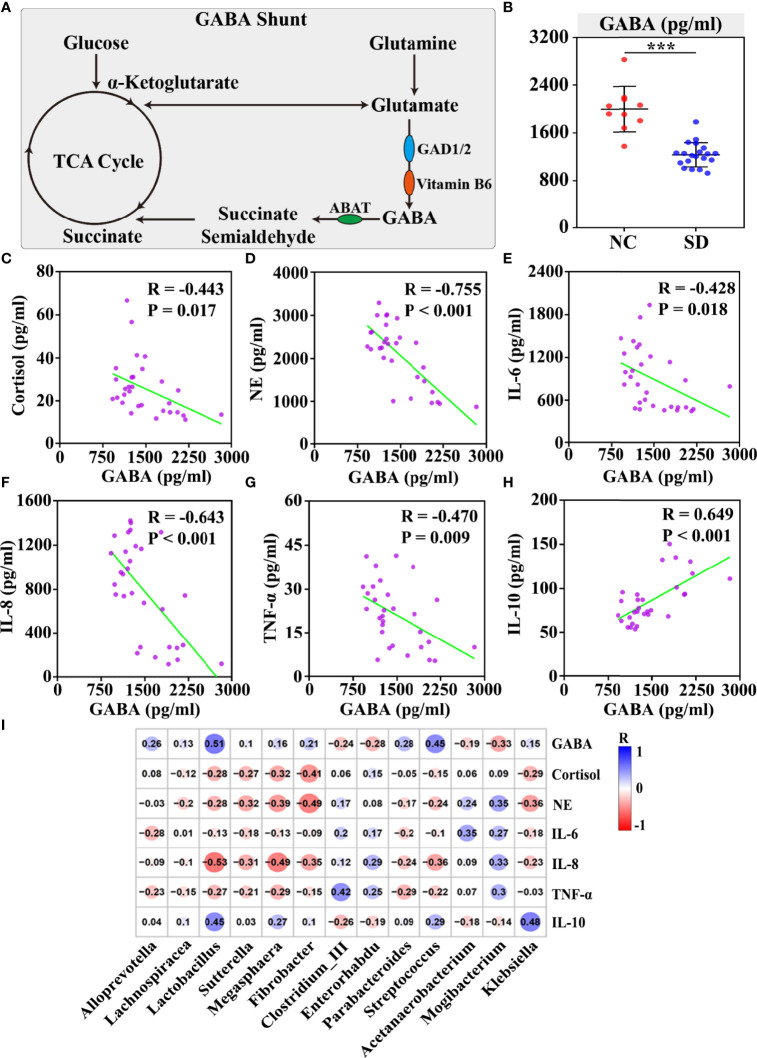
The correlation between plasma GABA and sleep deprivation (SD) altered stress hormones and gut microbiota disorders. **(A)** Schematic of the GABA shunt and related enzymes. **(B)** The concentration of serum GABA was measured by ELISAs. Plasma GABA was negatively correlated with cortisol **(C)**, norepinephrine **(D)**, IL-6 **(E)**, IL-8 **(F)**, and TNF-α **(G)**, but positively associated with the anti-inflammatory cytokine IL10 **(H)**. **(I)** The correlation between thirteen SD altered differential genera and plasma stress hormones and inflammatory cytokines was shown in the correlation matrix. The blue and red color indicated positive and negative correlation, respectively. The correlation strength was signified by color intensity. The greater the color intensity, the stronger the correlation. Differences were denoted as follows: ***p < 0.001. TCA cycle, tricarboxylic acid cycle; GAD, glutamate decarboxylase.

GABA is the primary inhibitive neurotransmitter of the central nervous system (CNS) and serves as an important mediator of the sleep/wake flip-flop cycle (21). Although GABA is maintained outside the brain by the blood–brain barrier (BBB) under normal conditions (22), some studies have revealed that both physical and psychological stress can exacerbate BBB permeability (23). *In vivo*, GABA is mainly produced from glutamate by glutamate decarboxylase (GAD) 1 and GAD2 with the assistance of Vitamin B6, and is catabolized by GABA-transaminase (**Figure 4A**).

In this study, we found that the differential gut microbiota and metabolites between the SD group and the control group are enriched in the signaling pathways related to GABA metabolism, including glutamate metabolism and Vitamin B6 metabolism. Moreover, the plasma concentration of GABA in the SD group was found to be markedly lower than that in the control group (*p* < 0.001) (**Figure 4B**). To investigate the role of plasma GABA in the consequences of SD, a correlation analysis was performed between plasma GABA and SD-induced stress responses and gut dysbiosis. We found that plasma GABA level was significantly associated with stress hormones and cytokines, including cortisol (*R* = −0.443, *p* < 0.05), NE (*R* = −0.755, *p* < 0.001), IL-6 (*R* = −0.428, *p* < 0.05), IL-8 (*R* = −0.643, *p* < 0.001), TNF-α (*R* = −0.470, *p* < 0.01), and IL-10 (*R* = 0.649, *p* < 0.001) (**Figures 4C–H**). In addition, we examined the correlation between plasma GABA and SD-induced differential gut microbiota. The results revealed that those bacteria (such as *Lactobacillus*, *Sutterella*, *Megasphaera*, *Fibrobacter*, and *Klebsiella*) that were negatively correlated with stress hormones and proinflammatory cytokines were significantly positively associated with plasma GABA level, but those (such as Clostridium_III and *Mogibacterium*) positively correlated with stress hormones and proinflammatory cytokines were found to be negatively associated with plasma GABA level (**Figure 4I**). It has been reported that the GABAergic projection pathway to hypothalamus paraventricular nucleus could inhibit the HPA axis (24). Therefore, we speculated that GABA might mediate SD-induced stress hormones and gut dysbiosis.

### Effect of Probiotics Supplementation on the Consequences of SD

*Lactobacillus* and *Bifidobacterium* are the key members of gut microbiota in the production of GABA (25–27). Therefore, we applied BTVC, containing *Bifidobacterium*, *Lactobacillus*, and *E. faecalis*, to examine the effect of gut-derived GABA on the consequences of SD. Because one stool sample in the control group is not qualified, a total of 13 stool samples were recruited in this analysis.

**Figure 5:**
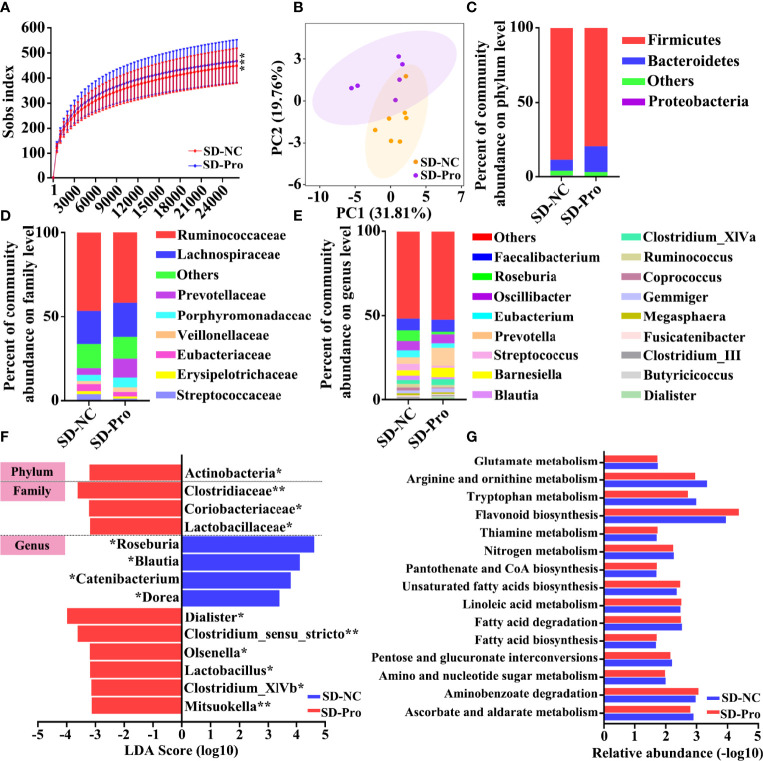
Composition of gut microbiota after probiotics supplementation in sleep deprivation (SD) rhesus monkeys (RMs). **(A)** Rarefaction curves for the gene number in the control (*n* = 7) and probiotics supplementation (*n* = 6) groups. **(B)** Principal coordinate analysis showed that SD RMs with probiotics supplementation were significantly different from those controls. The relative abundance of fecal bacteria at phylum **(C)**, family **(D)**, and genus **(E)** levels was clustered into different groups. Only phyla meeting relative abundances of more than 0.5% was recruited in this analysis. **(F)** The differential bacteria between probiotics and the control group were identified by LEfSe methods. The probiotics-enriched taxa were indicated with a negative LDA score (red), and the control enriched taxa were indicated with a positive LDA score (blue). **(G)** The fifteen differential KEGG pathways based on differential gut microbiota were shown. Differences were denoted as follows: *p < 0.05; **p < 0.01; ***p < 0.001. SD-NC, SD control group; SD-Pro, SD group with probiotics supplementation.

First, we assessed the effect of BTVC on long-term SD-induced gut flora disorders. A total of 833,027 high-quality sequences were identified (**Table S3**). Under the condition of sufficient sequencing depth, the rate of new gene acquisition in the BTVC group was obviously faster than that in the control group (**Figure 5A**). The β‐diversity analysis presented a distinct clustering between the control group and the BTVC group (**Figure 5B**). The supplementation of BTVC will greatly restore the relative abundance of intestinal microbiota at the phylum, family, and genus levels (**Figures 5C–E**). In addition, we found that the relative abundance of *Lactobacillus* was significantly increased after the supplementation with BTVC (**Figure 5F**). The KEGG results based on differential gut microbiota demonstrated that glutamate and tryptophan metabolism were involved in the process (**Figure 5G**).

**Figure 6:**
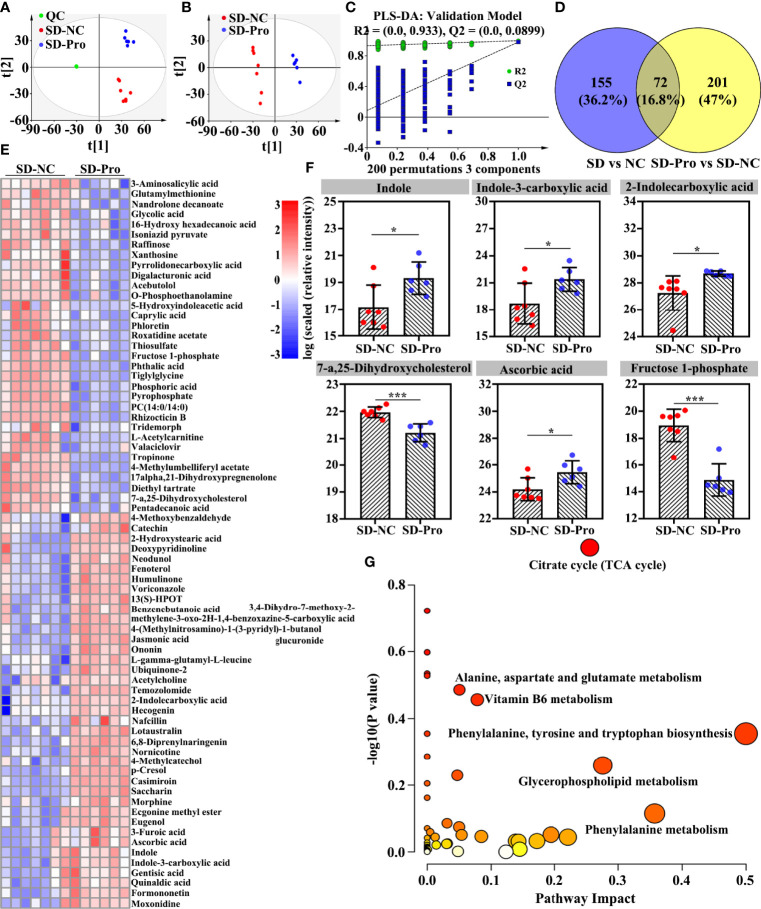
Effects of probiotics supplementation on sleep deprivation (SD) altered gut metabolites in rhesus monkeys (RMs). **(A)** SD RMs from the probiotics group were clearly separated from those controls in the PCA score plot (5 principal components, R2X = 0.837 and Q2 = 0.570). **(B)** Metabolites from the probiotics intervention group clustered well away from controls in the PLS-DA score plot (3 principal components, R2X = 0.610, R2Y = 0.999, and Q2 = 0.982). **(C)** The results of the PLS-DA model were validated by the permutation test [*n* = 200, R2 = (0.0, 0.933), and Q2 = (0.0, 0.0899)]. **(D)** The Venn diagram presented the overlap of differential metabolites between SD and probiotics altered metabolites. **(E)** Seventy-two gut differential metabolites were changed after probiotics supplementation. **(F)** The representative of gut differential metabolites was compared and shown using a box map. **(G)** The involved metabolic pathways following probiotics supplementation. The value of *p* and pathway impact is calculated from the pathway enrichment and topology analysis, respectively. The node color is based on its *p*-value and the node radius is determined based on its pathway impact values. Differences were denoted as follows: *p < 0.05; ***p < 0.001. SD-NC, SD control group; SD-Pro, SD group with probiotics supplementation; QC, quality control sample.

Similarly, a significant change was found in gut metabolite profiles after the supplementation with BTVC. A total of 273 differential metabolites were identified (**Table S4**). As shown in the PCA plot, SD RMs from the BTVC group were clearly separated from those of controls (**Figure 6A**). In addition, the PLS-DA score plot presented a distinct clustering between the control group and the BTVC group (**Figure 6B**). The results of the PLS-DA model were validated by the permutation test (*n* = 200, R2Y = 0.933, and Q2 = 0.090) (**Figure 6C**). Among the 227 differential metabolites caused by SD, 72 (31.7%) were found to be significantly changed after BTVC supplementation (**Figures 6D, E**), including indole, indole-3-carboxylic acid, 2-indolecarboxylic acid, 7-a,25-dihydroxycholesterol, ascorbic acid, and fructose 1-phosphate (**Figure 6F**). To further investigate the biological functions of these differential metabolites, pathway enrichment analysis was performed. The metabolic disorders mainly involved tricarboxylic cycle, glutamate metabolism, Vitamin B6 metabolism, and tryptophan metabolism (**Figure 6G**).

**Figure 7:**
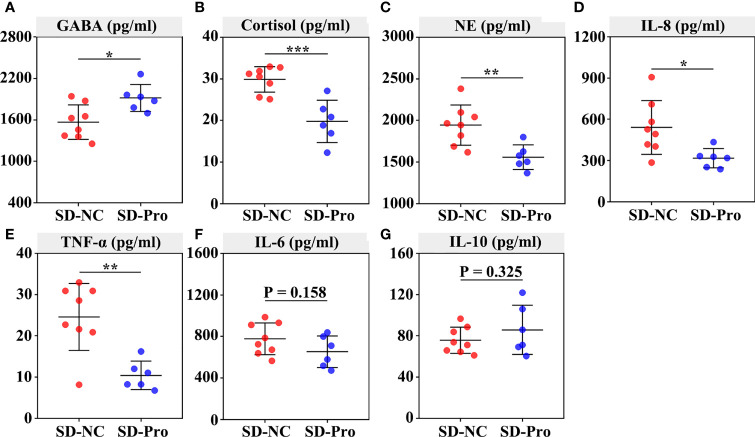
Effects of probiotics on the secretion of plasma GABA, stress hormones, and cytokines in rhesus monkeys. The plasma was extracted from the centrifuged blood samples after 30 days of probiotics supplementation. The concentration of serum GABA **(A)**, cortisol **(B)**, norepinephrine **(C)**, IL‐8 **(D)**, TNF‐α **(E)**, IL-6 **(F)** and IL‐10 **(G)** were detected using ELISA assays. Differences were denoted as follows: *P < 0.05; **P < 0.01; ***P < 0.001. Abbreviations: SD-NC, SD control group; SD-Pro, SD group with probiotics supplementation.

Finally, we examined the effect of BTVC on plasma GABA, stress hormones, and cytokines. The results showed that BTVC supplementation could significantly increase plasma GABA levels (*p* < 0.05) and reduce plasma cortisol (*p* < 0.001) and NE (*p* < 0.01) levels in SD RMs (**Figures 7A–C**). Additionally, the addition of BTVC greatly contributed to the reduction of proinflammatory cytokines IL-8 (*p* < 0.05) and TNF-α (*p* < 0.01) (**Figures 7D, E**), but 232 had no effect on the levels of IL-6 and IL-10 (**Figures 7F, G**). These results disclosed that BTVC supplementation could alleviate SD-induced stress responses and gut dysbiosis *via* increasing the relative abundance of *Lactobacillus* and plasma GABA concentration.

## Discussion

SD has become a health problem in the modern society. By definition, sleeping less than 6 h for six consecutive nights is considered chronic SD (28). In this study, we applied paradoxical sleep, bright light, and noise to build the RM SD model. The results of our study demonstrated that SD could give rise to the activation of stress responses, a reduction of gut microbiota diversity and richness, and the disorders of gut metabolites. Probiotics supplementation could markedly reverse SD-induced stress responses and gut dysbiosis by increasing the relative abundance of *Lactobacillus* and plasma GABA concentration.

Compulsive exercise (29) and water platform (30) are currently the most common methods to establish an SD mouse model. However, there is a lack of instruments on the market that can be directly used for SD in RM. Therefore, exploring appropriate methods to build an RM SD model is the first question to be addressed in this study. Paradoxical SD has been found to be associated with increased glucocorticoid levels and was regarded as a useful tool to study the impact of sleep disturbance (31, 32). Bunnell et al. reported that bright light could significantly suppress salivary melatonin and increase rapid eye movement latency and non-rapid eye movement period length (33). Muzet et al. reported that long-term exposure to environment noises could induce marked tiredness, increase low vigilance state, and reduce daytime performance (34). In this study, we applied paradoxical sleep, bright light, and noise to disrupt the sleep rhythm in RM.

As a strong endogenous stressor, SD could disrupt the intestinal barrier by activating the HPA axis and the SAM system. Numerous studies have reported that stress could drive systemic inflammatory cytokines’ upregulation by stimulating immune and inflammatory cells (5, 6). As an important component of intestinal barrier, the gut microbiota community was also found to be affected by stress responses. Gao et al. reported that excessive corticosterone is a core risk factor of gut microbiota disorders and colitis in SD mice (35). Song et al. found that norepinephrine could alter gut microbiota and induce neurotoxicity in α-synuclein mutant mice by depleting toxin DSP-4 and LPS (36). Consistent with these findings, our results showed that SD could lead to an increase of plasma stress hormones and proinflammatory cytokines, and gut microbiota and metabolite disorders in RMs. These results supported the idea that paradoxical sleep, bright light, and noises can be used for the establishment of an RM SD model.

Previous studies have demonstrated the role of melatonin in alleviating SD-induced gut microbiota disorders and intestinal barrier dysfunction (30, 35). Olivier et al. reported that gastrointestinal melatonin production was affected by its precursor tryptophan in stressed and non-stressed rainbow trout (37). In this study, we also found that the differential gut metabolites between the SD group and the control group were enriched in the process of tryptophan metabolism. In addition, we observed that GABA, an important inhibitive neurotransmitter in CNS, was correlated with SD-induced stress responses and gut dysbiosis. Some studies have revealed that the GABAergic projection pathway from the anterior lateral bed nucleus of stria terminalis to the paraventricular nucleus of hypothalamus can inhibit the activation of the HPA axis (24). Ide et al. also reported that central neuropeptide Y could significantly antagonize the overexpression of central CRH and attenuate the activation of the HPA axis through the GABAA receptor (38). Although GABA was maintained outside the brain by BBB under normal conditions (22), physical and psychological stress can increase BBB permeability and subsequent GABA influx (23).

*Lactobacillus* and *Bifidobacterium* are the key members of gut microbiota in the production of GABA (25–27). It has been reported that the acidic environment caused by *Bifidobacterium* and *Lactobacillus* is conducive to stimulate intestinal peristalsis, maintain intestinal physiological function, and increase the digestion and absorption of nutrients such as protein, lactose, calcium, and vitamins (39, 40). Therefore, BTVC was used to increase the relative abundance of gut GABA-producing microbiota and the absorption of gut-derived GABA in our study. The results of our study showed that BTVC supplementation only increased the relative abundance of *Lactobacillus*, but did not change *Bifidobacterium* or *Enterococcus*. The specific reasons behind this are still not understood. However, as we know, gut microbiota constitutes a complicated but manifold ecosystem, in which specific symbiotic or antagonistic relationships are formed among various bacteria (41). Therefore, we speculated that there might be some factors or flora that limit the growth of *Bifidobacterium* and *Enterococcus* during BTVC supplementation. In addition, we found that BTVC supplementation could significantly increase plasma GABA level and alleviate SD-induced stress responses and gut dysbiosis. Among the fourteen significantly changed flora caused by BTVC supplementation, only *Lactobacillus* has the ability to produce GABA. Based on the above results, we proposed that *Lactobacillus* is responsible for the increased level of plasma GABA and the amelioration of SD-induced stress responses and gut dysbiosis.

**Figure 8:**
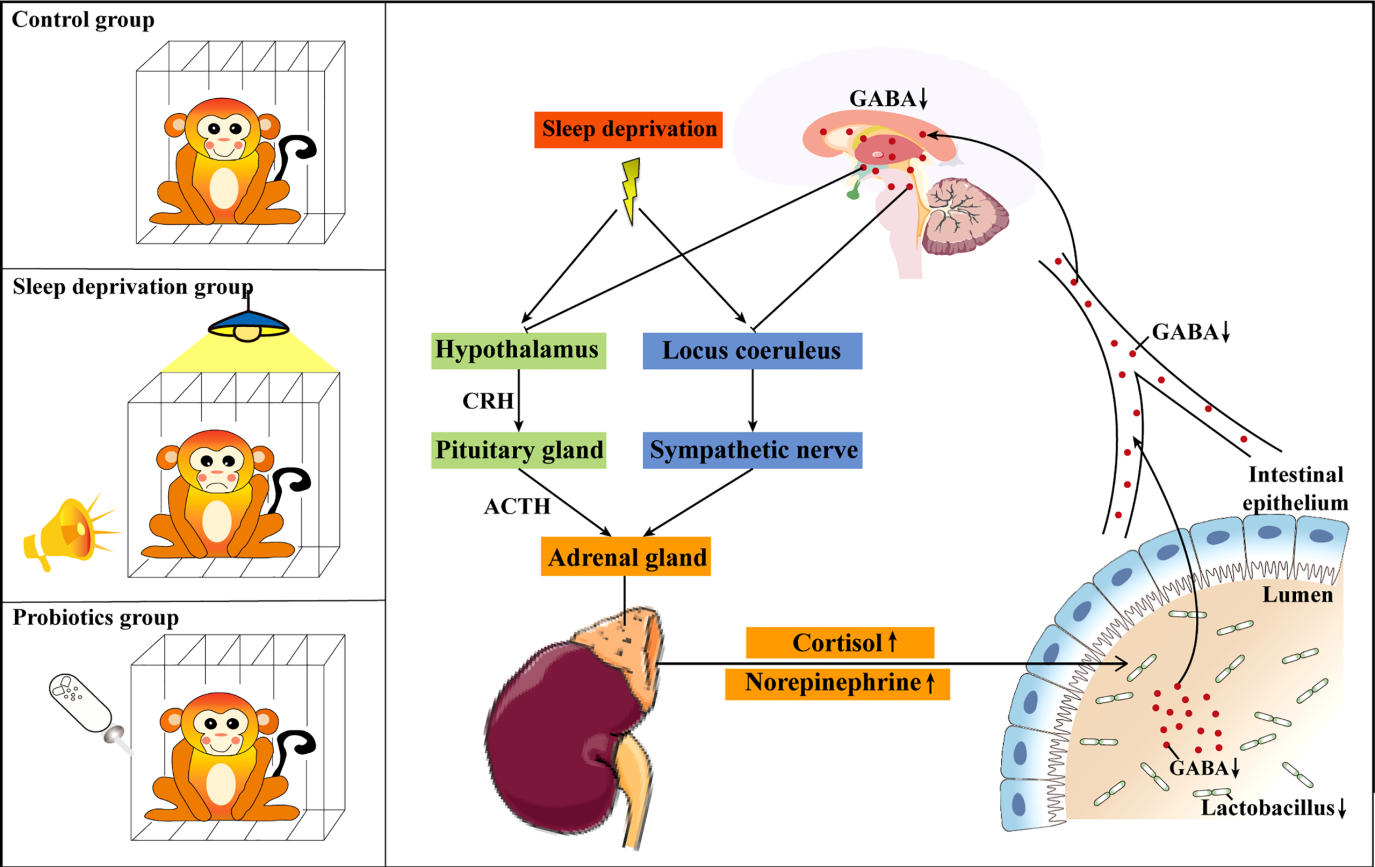
Schematic diagram of the possible mechanism of *Lactobacillus* ameliorating sleep deprivation (SD)-induced stress responses and gut dysbiosis. *Lactobacillus* alleviated SD-induced stress responses and gut dysbiosis by increasing the absorption of gut-derived GABA. CRH, corticotropin-releasing hormone; ACTH, adrenocorticotropic hormone.

In conclusion, the present study investigated the potential mechanism of SD-induced stress responses and gut dysbiosis in RMs. We were the first to confirm that paradoxical sleep, bright light, and noise can be used for the establishment of an RM SD model. In addition, we found that SD-induced stress response and gut dysbiosis might be an outcome of reduced gut-derived GABA absorption. The supplementation of *Lactobacillus* might be an effective treatment for the SD-induced intestinal dysfunction (**Figure 8**).

## Data Availability Statement

The datasets presented in this study can be found in online repositories. The name of the repository and accession number can be found below: EMBL-EBI MetaboLights; MTBLS4928.

## Ethics Statement

The animal study was reviewed and approved by The ethics committee of Tongji Medical College of Huazhong University of Science and Technology, Wuhan, China.

## Author Contributions

XS and JS conceived the study and supervised all aspects of the work. NZ, YS., and CJ designed the research, prepared the figures and tables, and wrote the paper. ZZ, HB, and XL performed the experiments and interpreted the data. XC and YZ contributed analytical tools. All authors read and approved the manuscript.

## Funding

This study was supported by the National Natural Science Foundation of China (NSFC) (Grant number: 82072744) and Fundamental Research Funds for the Central Universities (Grant number: 2021yjsCXCY111).

## Conflict of Interest

The authors declare that the research was conducted in the absence of any commercial or financial relationships that could be construed as a potential conflict of interest.

## Publisher’s Note

All claims expressed in this article are solely those of the authors and do not necessarily represent those of their affiliated organizations, or those of the publisher, the editors and the reviewers. Any product that may be evaluated in this article, or claim that may be made by its manufacturer, is not guaranteed or endorsed by the publisher.

## Supplementary Material

Supplementary Figure 1 Gut microbiota of sleep‐deprived rhesus monkey differs from those controls. **(A)** The Venn diagram illustrates the overlap of OTUs in the gut microbiota between these two groups. **(B, C)** The relative abundance of the fecal bacterial family and genera was clustered into different groups, and the microbiota compositions significantly differed. In this analysis, only communities with relative abundances of greater than 0.5% were included. All OTUs with lower abundances were grouped as “others”.
