# Supplementary figures and images for: Lactobacillus Ameliorates SD-Induced Stress Responses and Gut Dysbiosis by Increasing the Absorption of Gut-Derived GABA in Rhesus Monkeys
Source: Front Immunol. 2022 Jul 7;13:915393. doi: 10.3389/fimmu.2022.915393 (PMC9302489; doi:10.3389/fimmu.2022.915393)

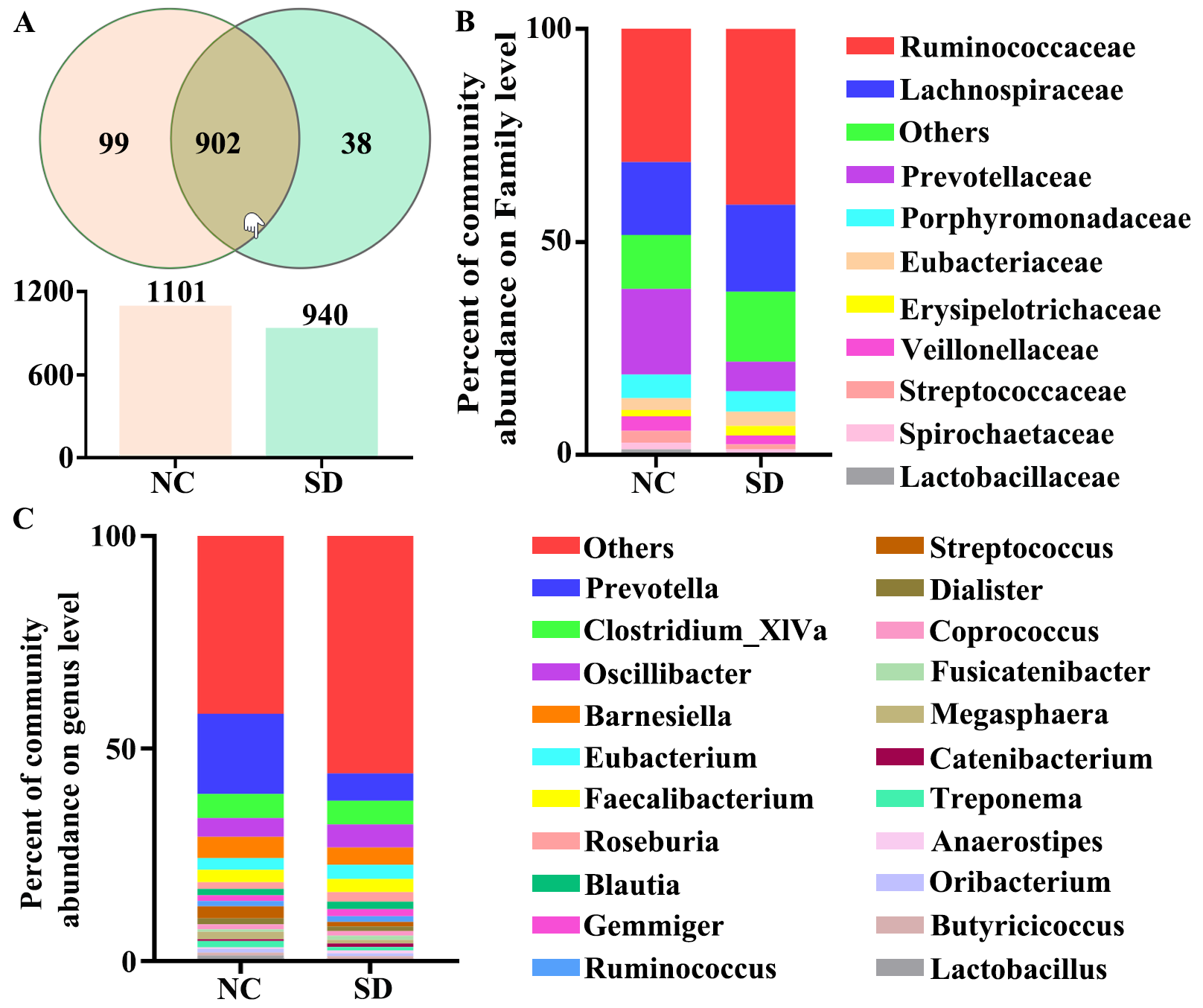

Supplement: Supplementary Figure 1 — Gut microbiota of sleep‐deprived rhesus monkey differs from those controls. (A) The Venn diagram illustrates the overlap of OTUs in the gut microbiota between these two groups. (B, C) The relative abundance of the fecal bacterial family and genera was clustered into different groups, and the microbiota compositions significantly differed. In this analysis, only communities with relative abundances of greater than 0.5% were included. All OTUs with lower abundances were grouped as “others”. [file Image_1.tif]
