# Supplementary material for: Lactobacillus Ameliorates SD-Induced Stress Responses and Gut Dysbiosis by Increasing the Absorption of Gut-Derived GABA in Rhesus Monkeys
Source: Front Immunol. 2022 Jul 7;13:915393. doi: 10.3389/fimmu.2022.915393 (PMC9302489; doi:10.3389/fimmu.2022.915393)
Supplement: Supplementary file 2 [file Table_1.docx]

Table S1. Comparison of phylotype coverage and diversity estimation of the 16S rRNA gene libraries between sleep deprivation and control group.

| sample | No. of reads | No. of OTUs | Good’s coverage | Chao | Shannon | ACE | Simpson |
| --- | --- | --- | --- | --- | --- | --- | --- |
| NC01 | 68081 | 548 | 0.997 | 641.80 | 4.71 | 641.14 | 0.019 |
| NC02 | 68788 | 499 | 0.997 | 588.83 | 3.79 | 584.51 | 0.088 |
| NC03 | 68267 | 607 | 0.997 | 674.78 | 4.26 | 682.31 | 0.053 |
| NC04 | 67793 | 579 | 0.998 | 648.49 | 4.47 | 654.83 | 0.039 |
| NC05 | 68787 | 591 | 0.997 | 678.55 | 4.78 | 657.94 | 0.020 |
| NC06 | 67877 | 594 | 0.997 | 715.69 | 4.52 | 678.55 | 0.034 |
| NC07 | 68074 | 566 | 0.997 | 626.76 | 4.74 | 628.02 | 0.020 |
| NC08 | 68573 | 585 | 0.997 | 666.18 | 4.80 | 651.17 | 0.017 |
| NC09 | 68236 | 613 | 0.997 | 700.25 | 4.86 | 694.78 | 0.017 |
| NC10 | 67679 | 572 | 0.997 | 654.69 | 4.69 | 641.28 | 0.022 |
| SD01 | 68952 | 477 | 0.997 | 578.00 | 4.46 | 576.75 | 0.024 |
| SD02 | 68336 | 499 | 0.998 | 549.71 | 4.68 | 549.17 | 0.018 |
| SD03 | 67807 | 508 | 0.997 | 638.78 | 4.46 | 623.24 | 0.025 |
| SD04 | 68060 | 563 | 0.997 | 646.90 | 4.80 | 647.78 | 0.017 |
| SD05 | 68383 | 602 | 0.997 | 696.45 | 4.75 | 692.49 | 0.020 |
| SD06 | 68234 | 594 | 0.997 | 701.61 | 4.50 | 663.31 | 0.041 |
| SD07 | 68242 | 533 | 0.997 | 623.37 | 4.62 | 619.25 | 0.019 |
| SD08 | 68138 | 485 | 0.997 | 600.56 | 4.40 | 595.42 | 0.027 |
| SD09 | 67883 | 610 | 0.997 | 706.30 | 4.78 | 696.49 | 0.018 |
| SD10 | 68506 | 532 | 0.997 | 617.13 | 4.68 | 602.23 | 0.020 |
| SD11 | 68783 | 593 | 0.998 | 651.04 | 4.68 | 655.40 | 0.022 |
| SD12 | 67505 | 519 | 0.997 | 607.39 | 4.51 | 598.79 | 0.025 |
| SD13 | 68270 | 575 | 0.997 | 652.34 | 4.56 | 647.44 | 0.023 |
| SD14 | 68659 | 493 | 0.997 | 582.68 | 4.23 | 577.79 | 0.044 |
| SD15 | 68040 | 587 | 0.997 | 677.02 | 4.49 | 669.40 | 0.037 |
| SD16 | 68638 | 549 | 0.997 | 654.47 | 4.49 | 634.21 | 0.028 |
| SD17 | 68194 | 457 | 0.998 | 544.57 | 4.49 | 521.38 | 0.025 |
| SD18 | 68779 | 530 | 0.997 | 599.49 | 4.62 | 607.81 | 0.022 |
| SD19 | 69203 | 490 | 0.997 | 609.02 | 4.28 | 585.45 | 0.034 |
| SD20 | 67678 | 421 | 0.998 | 496.48 | 3.59 | 486.54 | 0.081 |
