# Supplementary material for: Lactobacillus Ameliorates SD-Induced Stress Responses and Gut Dysbiosis by Increasing the Absorption of Gut-Derived GABA in Rhesus Monkeys
Source: Front Immunol. 2022 Jul 7;13:915393. doi: 10.3389/fimmu.2022.915393 (PMC9302489; doi:10.3389/fimmu.2022.915393)
Supplement: Supplementary file 3 [file Table_2.docx]

Table S2. The significantly differential metabolites between sleep-deprivation rhesus monkeys and controls.

| Name | VIP | P Value | Log(FC) |
| --- | --- | --- | --- |
| (1R,2R)-3-[(1,2-Dihydro-2-hydroxy-1-naphthalenyl)thio]-2-oxopropanoic acid | 1.86172 | 0.000574 | -1.58785 |
| (R)-Oxypeucedanin | 1.67243 | 0.001274 | -1.37172 |
| (S,E)-Zearalenone | 1.01126 | 0.009158 | 0.553846 |
| (Z)-9-Cycloheptadecen-1-one | 1.01996 | 0.037229 | 0.435935 |
| 1,9-Nonanedithiol | 1.34589 | 0.000187 | 0.711472 |
| 13(S)-HPOT | 1.36657 | 0.025619 | 1.234448 |
| 16-Hydroxy hexadecanoic acid | 1.19473 | 0.010775 | 0.71924 |
| 17alpha,21-Dihydroxypregnenolone | 2.48618 | 0.000111 | 2.719343 |
| 17-Hydroxyprogesterone | 1.2653 | 0.023244 | -0.91819 |
| 1H-Indole-3-acetamide | 2.14953 | 0.032013 | -2.15632 |
| 2,6-Di-tert-butyl-4-methylphenol | 1.28626 | 0.005281 | -0.81622 |
| 2-Furoic acid | 1.08111 | 0.008868 | -0.55476 |
| 2-Hydroxystearic acid | 1.5423 | 0.001951 | 1.22009 |
| 2-Indolecarboxylic acid | 1.46913 | 0.024171 | -1.31332 |
| 2-Methylfuran | 1.41235 | 0.020677 | -1.10005 |
| 2-Oxoarginine | 1.65159 | 0.019899 | -1.56603 |
| 2-Undecyl-4(1H)-quinolinone | 1.38306 | 0.012863 | 1.216099 |
| 3-(3,4,5-Trimethoxyphenyl)propanoic acid | 1.40231 | 0.007188 | -1.11498 |
| 3, 5-Tetradecadiencarnitine | 1.18699 | 0.013278 | -0.79207 |
| 3,4-Dihydro-7-methoxy-2-methylene-3-oxo-2H-1,4-benzoxazine-5-carboxylic acid | 1.83341 | 0.021321 | -1.79548 |
| 3,4-Dihydroxyhydrocinnamic acid | 1.04929 | 0.010001 | 0.600853 |
| 3-Aminosalicylic acid | 1.30255 | 0.018857 | -1.02735 |
| 3-Furoic acid | 1.51799 | 0.021954 | -1.56957 |
| 3-Hydroxylidocaine | 1.26459 | 0.000815 | 0.720253 |
| 3-Methylcrotonylglycine | 1.94845 | 0.00505 | -1.98925 |
| 3-Succinoylpyridine | 1.367 | 0.001559 | 0.891713 |
| 4-(2-Aminophenyl)-2,4-dioxobutanoic acid | 1.94744 | 0.007545 | -1.90823 |
| 4-(Methylnitrosamino)-1-(3-pyridyl)-1-butanol glucuronide | 1.23532 | 0.044059 | 1.102719 |
| 4',5,7-Trimethoxyflavone | 1.35141 | 0.000451 | 0.804873 |
| 4-Acetamidobutanoic acid | 1.23304 | 0.000442 | 0.600338 |
| 4-Bromophenol-2,3-epoxide | 1.33326 | 0.031197 | -1.03667 |
| 4-Hydroxybenzaldehyde | 1.75282 | 0.027977 | -1.63332 |
| 4-Hydroxyphenylpyruvic acid | 1.61267 | 0.007792 | -1.30839 |
| 4-Methoxybenzaldehyde | 1.04586 | 0.043194 | 0.610145 |
| 4-Methylcatechol | 1.29601 | 0.022395 | 1.010351 |
| 4-Methylumbelliferyl acetate | 1.89911 | 0.008493 | -1.89678 |
| 4-Nitrophenol | 1.44348 | 0.001629 | -0.93573 |
| 5-Hydroxyindoleacetic acid | 1.50874 | 0.015564 | -1.22669 |
| 6,8-Diprenylnaringenin | 1.0437 | 0.030622 | -0.63853 |
| 6-Hydroxynicotinic acid | 2.09721 | 1.90E-05 | -1.74711 |
| 6-Methylthiopurine 5'-monophosphate ribonucleotide | 1.89065 | 0.021391 | -1.86737 |
| 7-a,25-Dihydroxycholesterol | 1.02822 | 0.021061 | 0.597418 |
| 7-Methylxanthine | 1.2303 | 0.004015 | 0.682181 |
| 8-Hydroxy-7-methylguanine | 1.68319 | 0.011991 | -1.45016 |
| Acebutolol | 1.00768 | 0.042741 | 0.620455 |
| Acetaminophen | 1.35117 | 0.012701 | -0.9446 |
| Acetylcholine | 2.73629 | 0.047311 | 3.866148 |
| Acevaltrate | 1.75436 | 0.038909 | -1.71794 |
| Aflatoxin B2 | 1.25475 | 0.024066 | -1.04682 |
| Aliskiren | 1.73058 | 0.008555 | 1.193804 |
| Amoxapine | 1.08658 | 0.0018 | 0.474779 |
| Arginyl-Proline | 2.0469 | 0.0292 | -1.31191 |
| Ascorbic acid | 1.78802 | 0.010945 | -1.98629 |
| Avocadyne 1-acetate | 1.56394 | 0.003075 | -1.13431 |
| Benzenebutanoic acid | 1.15598 | 0.002782 | 0.632579 |
| Betulin | 1.14532 | 0.002937 | 0.588283 |
| Bluensidine | 1.172 | 0.04075 | -0.76078 |
| Caprylic acid | 1.66926 | 1.95E-05 | 1.073195 |
| Capryloylglycine | 1.26355 | 0.025331 | -1.08042 |
| Carbidopa | 1.35042 | 0.006948 | -1.00318 |
| Carbinoxamine | 1.6725 | 0.002253 | -1.37903 |
| Casimiroin | 1.49835 | 0.037467 | -1.4011 |
| Catechin | 1.89972 | 0.007672 | -1.68 |
| Ceramide (d18:1/16:0) | 1.49414 | 0.025202 | 0.826246 |
| Chloropyramine | 1.68749 | 0.000857 | -1.15374 |
| Cholic acid | 1.09075 | 0.017282 | -0.72565 |
| Cinnamoylglycine | 2.7855 | 0.000405 | -3.61784 |
| Cortisol | 1.1637 | 0.029912 | -0.76176 |
| Cotinine N-oxide | 1.98425 | 0.005613 | -2.29983 |
| Creatine | 1.67619 | 0.03874 | 1.749238 |
| Cucurbitacin S | 1.09576 | 0.010918 | 0.623536 |
| Dambonitol | 1.45141 | 0.003586 | 1.084803 |
| Demethylated antipyrine | 1.69728 | 0.004068 | -1.41944 |
| Deoxypyridinoline | 1.5605 | 0.022228 | 1.553792 |
| Deoxyuridine | 1.18578 | 0.049636 | 0.868977 |
| Diethyl tartrate | 1.23948 | 0.007206 | 0.814837 |
| Digalacturonic acid | 1.44582 | 0.015657 | -1.16738 |
| Digitogenin | 1.8354 | 0.001108 | 1.639378 |
| Diosmetin | 1.07199 | 0.049023 | -0.8656 |
| D-Maltose | 1.70242 | 0.00232 | -1.19641 |
| Docosanamide | 1.55388 | 0.045897 | 1.6253 |
| Dodecanoylcarnitine | 1.08392 | 0.033351 | 0.516905 |
| Ecgonine | 1.16391 | 0.030547 | -0.73449 |
| Ecgonine methyl ester | 1.12202 | 0.021757 | -0.73469 |
| Enalapril | 1.69055 | 0.024402 | -1.77055 |
| Erlotinib | 1.37112 | 0.000173 | 0.754495 |
| Ethoxyquin | 1.41942 | 0.005737 | 0.974324 |
| Ethyl cinnamate | 1.85573 | 3.94E-06 | 1.258352 |
| Eugenitin | 1.91284 | 1.79E-06 | 1.310118 |
| Eugenol | 1.00908 | 0.034386 | -0.69769 |
| Fenoterol | 1.3607 | 0.001868 | 0.827742 |
| Ferulic acid 4-sulfate | 1.42369 | 0.010952 | -1.05567 |
| Floionolic acid | 1.32413 | 0.005391 | -0.97489 |
| Formononetin | 2.07356 | 0.000133 | -1.90988 |
| Fortimicin FU-10 | 2.33296 | 0.003393 | -2.09753 |
| Fructose 1-phosphate | 1.90473 | 0.006071 | 1.798746 |
| Furfuryl thioacetate | 1.83236 | 0.041007 | 1.619177 |
| Gamma-Tocopherol | 1.07745 | 0.005944 | 0.608647 |
| Gentisic acid | 1.45633 | 0.014324 | -1.05167 |
| Gibberellin A12 | 1.26794 | 0.018257 | 0.838604 |
| Glutamylmethionine | 1.01061 | 0.026273 | -0.59696 |
| Glycitein | 1.64225 | 0.011837 | -1.66567 |
| Glycolic acid | 1.10336 | 0.017386 | 0.670955 |
| Hecogenin | 1.86819 | 0.00344 | -1.9064 |
| Heptanoic acid | 1.92298 | 4.69E-06 | 1.398424 |
| Hexanoylglycine | 1.51714 | 0.013832 | -1.18526 |
| Hippuric acid | 2.40848 | 0.000113 | -2.57235 |
| Homovanillic acid sulfate | 1.35441 | 0.028299 | -1.05395 |
| Humulinone | 1.44416 | 0.000972 | 0.875477 |
| Hypusine | 1.52906 | 0.036393 | 1.530452 |
| Indole | 1.80112 | 0.02825 | -1.43451 |
| Indole-3-carboxylic acid | 1.92815 | 0.030261 | -1.64817 |
| Indoleacetic acid | 1.32244 | 0.015066 | -1.0391 |
| Indoxyl sulfate | 2.50898 | 0.014959 | -2.94171 |
| Inosine | 1.68357 | 0.012393 | 1.510209 |
| Iprobenfos | 1.32273 | 0.041838 | 1.153197 |
| Isoniazid pyruvate | 1.19571 | 0.031828 | 1.028699 |
| Isoquinoline | 1.32944 | 0.010766 | -1.02874 |
| Isorenieratene | 2.83863 | 0.000889 | -4.0591 |
| Jasmonic acid | 1.00689 | 0.010366 | 0.537109 |
| Ketorolac | 1.53037 | 0.000103 | 0.925936 |
| L-Acetylcarnitine | 1.04296 | 0.03184 | 0.646168 |
| L-Cystathionine | 1.95604 | 0.01513 | -1.87835 |
| Leucyl-Tryptophan | 1.30445 | 0.012819 | 1.025893 |
| Leucyl-Valine | 1.23862 | 0.043371 | 1.013087 |
| L-gamma-glutamyl-L-leucine | 1.41426 | 0.004668 | -0.95936 |
| L-Glutamic acid | 2.91528 | 0.016699 | 4.131204 |
| L-Hexanoylcarnitine | 1.72728 | 2.93E-05 | 1.184753 |
| Lisinopril | 1.19027 | 0.011683 | -0.8377 |
| L-Lysine | 2.15472 | 0.009113 | 2.733943 |
| Lotaustralin | 1.0735 | 0.015247 | -0.51785 |
| L-Thyronine | 1.59985 | 5.63E-05 | 1.025302 |
| Lysyl-Threonine | 1.61146 | 0.024259 | -1.40442 |
| Mandelic acid | 1.27404 | 0.047343 | -1.04933 |
| Meprobamate | 1.23295 | 0.035746 | 0.862257 |
| Mesobilirubinogen | 1.23756 | 0.017895 | 0.975877 |
| Mesoridazine | 1.15894 | 0.013973 | 0.732504 |
| Mitragynine | 1.26716 | 0.012062 | 0.96913 |
| Mollicellin B | 1.51241 | 0.011474 | -1.27296 |
| Morphine | 1.92272 | 0.002448 | 1.866954 |
| Moschamine | 1.96269 | 0.000804 | 1.809458 |
| Moxonidine | 1.93215 | 0.002539 | -1.88137 |
| Myristic acid | 1.30119 | 0.002004 | 0.685441 |
| N2,N2-Dimethylguanosine | 1.28333 | 0.002428 | 0.809829 |
| N-acetyl-5-aminosalicylic acid | 1.17682 | 0.018157 | -0.84028 |
| N-Acetylarylamine | 1.98544 | 0.011803 | -2.32423 |
| N-Acetylhistidine | 1.47547 | 0.034937 | -1.19068 |
| N-Acetylleucine | 1.78402 | 0.016336 | 1.769493 |
| N-Acetyl-L-methionine | 1.15156 | 0.026028 | -0.63642 |
| N-Acetylneuraminate | 1.37822 | 0.001322 | -0.74073 |
| N-Acetylvanilalanine | 1.55943 | 4.98E-05 | 0.938373 |
| Nafcillin | 1.19457 | 0.008373 | -0.81372 |
| Nandrolone decanoate | 1.04836 | 0.03562 | 0.675383 |
| Naringin | 1.3052 | 0.012632 | 0.961403 |
| N-Benzoylaspartic acid | 1.30735 | 0.000378 | 0.726878 |
| Neodunol | 1.40961 | 0.001778 | 0.954655 |
| Neosaxitoxin | 1.36435 | 0.000309 | 0.796662 |
| Nicotinuric acid | 1.77563 | 0.03955 | -1.56518 |
| N-Lauroylglycine | 2.33096 | 0.006287 | -3.20994 |
| Nopaline | 1.10431 | 0.010499 | -0.63915 |
| Norepinephrine sulfate | 2.1231 | 0.005029 | -2.2042 |
| Norfuraneol | 1.03146 | 0.044285 | -0.46467 |
| Nornicotine | 1.55371 | 0.003115 | 1.295826 |
| Ononin | 1.18863 | 0.030642 | 0.526813 |
| O-Phosphoethanolamine | 1.36963 | 0.043542 | 0.867999 |
| Paracetamol sulfate | 2.22398 | 0.006978 | -2.42772 |
| PC(14:0/14:0) | 1.35689 | 0.013129 | 1.123543 |
| p-Cresol | 1.59416 | 0.008612 | -1.37545 |
| p-Cresol sulfate | 2.08882 | 0.022135 | -2.07655 |
| Pentadecanoic acid | 1.20164 | 0.009848 | 0.704766 |
| Phenprocoumon | 1.17123 | 0.002361 | 0.599983 |
| Phentolamine | 1.26 | 0.029489 | 1.113383 |
| Phenylacetylglycine | 1.39458 | 0.047058 | -1.02019 |
| Phloretin | 1.54875 | 0.016079 | -1.38861 |
| Phosphoric acid | 2.20244 | 0.006254 | 2.482861 |
| Phthalic acid | 1.26673 | 0.010705 | 0.919091 |
| p-Hydroxyphenylacetic acid | 1.81588 | 0.01245 | -1.78035 |
| Prazepam | 1.92929 | 0.009061 | -1.33133 |
| Probenecid | 1.3895 | 0.044373 | -1.27447 |
| Prolyl-Glutamine | 1.17123 | 0.010245 | -0.74804 |
| Propofol | 1.07122 | 0.045489 | -0.64369 |
| Prostaglandin E2 | 1.16236 | 0.020602 | 0.715463 |
| Pyridoxal | 1.20523 | 0.007451 | 0.513893 |
| Pyridoxine | 1.07945 | 0.009183 | 0.618338 |
| Pyroglutamic acid | 1.36341 | 0.044962 | -1.26291 |
| Pyrophosphate | 2.37825 | 0.005548 | 2.939189 |
| Pyrrolidonecarboxylic acid | 1.23699 | 0.010725 | -0.86755 |
| Quinaldic acid | 1.33383 | 0.024705 | -0.99343 |
| Quinoline | 1.43447 | 0.001981 | -1.01618 |
| Raffinose | 1.77567 | 0.001168 | -1.37997 |
| Rhizocticin B | 2.71832 | 0.000495 | 3.515541 |
| Roxatidine acetate | 1.37747 | 0.00838 | 1.115579 |
| Saccharin | 1.537 | 0.030133 | -1.32465 |
| Salicylic acid | 1.94005 | 0.000198 | -1.652 |
| Salicyluric acid | 1.90491 | 0.014443 | -1.73967 |
| Sanguinarine | 1.96361 | 0.017621 | -1.87099 |
| Scilliroside | 1.92131 | 0.002472 | 1.842396 |
| Sucrose | 1.94253 | 0.000391 | -1.40841 |
| Tacrine | 1.35599 | 0.012153 | -1.09869 |
| Taurine | 2.05851 | 0.010945 | -2.1633 |
| Temozolomide | 1.1636 | 0.005811 | 0.470194 |
| Terbutaline | 1.69015 | 0.005585 | -1.60791 |
| Testosterone sulfate | 2.02534 | 0.01263 | -2.21399 |
| Thiosulfate | 1.77222 | 0.003616 | 1.709713 |
| Tiglylcarnitine | 1.47386 | 0.018958 | 1.000756 |
| Tiglylglycine | 1.13004 | 0.019411 | 0.73965 |
| Tramadol | 1.29104 | 0.042783 | 0.97 |
| Tranexamic Acid | 1.16381 | 0.046315 | 0.792985 |
| Tricin | 1.37391 | 0.004366 | 0.921815 |
| Tridecanoic acid | 1.31949 | 0.00394 | 0.762052 |
| Tridemorph | 1.39329 | 0.013554 | 1.085672 |
| Trihexyphenidyl | 1.53736 | 0.021432 | 1.590179 |
| Tropinone | 1.56389 | 0.006555 | 1.345941 |
| Ubiquinone-2 | 1.16374 | 0.026399 | -0.84915 |
| Ubiquinone-4 | 1.35491 | 0.016655 | 1.09035 |
| Uridine | 1.9864 | 0.007572 | 1.810691 |
| Uridine 5'-monophosphate | 1.60304 | 0.000681 | 1.044008 |
| Valaciclovir | 1.50442 | 0.027251 | 1.173353 |
| Valyl-Valine | 1.39028 | 0.038642 | 1.119366 |
| Vanilloloside | 1.39908 | 0.009086 | -1.06452 |
| Voriconazole | 1.86987 | 0.008701 | 1.817295 |
| Wax ester | 1.34494 | 0.004462 | 0.831149 |
| Xanthosine | 2.01082 | 0.015617 | 2.260505 |
| Yangonin | 1.9554 | 0.00019 | -1.72052 |
| Zalcitabine | 1.37578 | 0.003659 | -0.95653 |
| Zeranol | 1.46557 | 0.006721 | 1.101874 |
| Zolmitriptan | 1.14579 | 0.001467 | 0.581857 |
