# Supplementary material for: Lactobacillus Ameliorates SD-Induced Stress Responses and Gut Dysbiosis by Increasing the Absorption of Gut-Derived GABA in Rhesus Monkeys
Source: Front Immunol. 2022 Jul 7;13:915393. doi: 10.3389/fimmu.2022.915393 (PMC9302489; doi:10.3389/fimmu.2022.915393)
Supplement: Supplementary file 4 [file Table_3.docx]

Table S3. Comparison of phylotype coverage and diversity estimation of the 16S rRNA gene libraries between probiotics and control group.

| sample | No. of reads | No. of OTUs | Good’s coverage | Chao | Shannon | ACE | Simpson |
| --- | --- | --- | --- | --- | --- | --- | --- |
| SD_NC01 | 67767 | 517 | 0.997 | 614.19 | 4.27 | 596.73 | 0.039 |
| SD_NC02 | 68443 | 468 | 0.997 | 552.06 | 4.39 | 554.84 | 0.027 |
| SD_NC03 | 67981 | 463 | 0.997 | 539.56 | 4.37 | 540.58 | 0.028 |
| SD_NC04 | 68262 | 469 | 0.998 | 615.45 | 4.19 | 581.33 | 0.031 |
| SD_NC05 | 14642 | 343 | 0.997 | 428.02 | 3.75 | 489.42 | 0.057 |
| SD_NC06 | 69139 | 585 | 0.997 | 667.40 | 4.63 | 663.71 | 0.022 |
| SD_NC07 | 67451 | 524 | 0.997 | 643.90 | 4.35 | 621.76 | 0.035 |
| SD_Pro01 | 67987 | 605 | 0.997 | 704.19 | 4.58 | 681.75 | 0.028 |
| SD_Pro02 | 67564 | 472 | 0.998 | 547.62 | 4.42 | 536.62 | 0.031 |
| SD_Pro03 | 68057 | 409 | 0.999 | 473.10 | 3.84 | 471.20 | 0.047 |
| SD_Pro04 | 68569 | 616 | 0.997 | 695.13 | 4.62 | 697.50 | 0.025 |
| SD_Pro05 | 68629 | 492 | 0.998 | 565.10 | 4.26 | 556.09 | 0.050 |
| SD_Pro06 | 68536 | 449 | 0.998 | 528.46 | 4.49 | 530.25 | 0.024 |
