# Supplementary material for: Lactobacillus Ameliorates SD-Induced Stress Responses and Gut Dysbiosis by Increasing the Absorption of Gut-Derived GABA in Rhesus Monkeys
Source: Front Immunol. 2022 Jul 7;13:915393. doi: 10.3389/fimmu.2022.915393 (PMC9302489; doi:10.3389/fimmu.2022.915393)
Supplement: Supplementary file 5 [file Table_4.docx]

Table S4. The significantly differential metabolites between sleep-deprivation rhesus monkeys with and without probiotics supplementation.

| Name | VIP | P Value | Log(FC) |
| --- | --- | --- | --- |
| (2S)-Liquiritigenin | 2.30172 | 0.002737 | 3.302218 |
| (5R)-5-Hydroxyhexanoic acid | 2.02683 | 0.002619 | -2.5709 |
| (R)-Heraclenol | 1.19182 | 0.000945 | 0.800604 |
| (R)-Salsolinol | 1.69151 | 3.13E-05 | 1.466438 |
| 100-1 | 1.78529 | 0.005029 | 2.09099 |
| 10-Nitrolinoleic acid | 1.45559 | 0.000108 | 1.113611 |
| 13(S)-HPOT | 1.52667 | 0.000279 | 1.278585 |
| 16-Hydroxy hexadecanoic acid | 1.28709 | 0.001095 | -0.95649 |
| 17alpha,21-Dihydroxypregnenolone | 2.33573 | 9.22E-07 | -2.65448 |
| 1-palmitoylglycerone 3-phosphate | 1.18233 | 0.02865 | 1.091888 |
| 1-Phenyl-1,3-octadecanedione | 2.68127 | 3.43E-06 | -3.55649 |
| 2-(3,4-dihydroxyphenyl)-3,4-dihydro-2H-1-benzopyran-3,5,7-triol | 1.38156 | 0.000201 | 1.005995 |
| 2,3-Dihydroxyindole | 1.57488 | 0.045032 | 2.011299 |
| 2,5-Dimethyl-4-ethoxy-3(2H)-furanone | 2.64521 | 4.31E-05 | 3.672806 |
| 2,6-diaminohexanoic acid | 1.59358 | 5.28E-05 | 1.315478 |
| 2-Amino-4-nitrotoluene | 2.82038 | 5.5E-10 | -3.6705 |
| 2-Aminoheptanedioic acid | 1.15916 | 8.67E-05 | -0.66133 |
| 2'-Hydroxy-4,4',6'-trimethoxychalcone | 1.34023 | 8.18E-05 | 0.910419 |
| 2-Hydroxystearic acid | 1.63502 | 0.000355 | 1.490248 |
| 2-Indolecarboxylic acid | 1.44563 | 0.012135 | 1.440006 |
| 2-Methylcitric acid | 1.87752 | 1.4E-05 | -1.78509 |
| 3-(2-Hydroxyphenyl)propanoic acid | 1.88399 | 3.19E-07 | 1.689773 |
| 3-(4-Methoxyphenyl)-2-propenal | 1.04805 | 0.000371 | 0.554312 |
| 3,4-Dihydro-7-methoxy-2-methylene-3-oxo-2H-1,4-benzoxazine-5-carboxylic acid | 1.98253 | 0.000222 | 2.162416 |
| 3,4-Dimethylbenzoic acid | 1.4631 | 4.06E-05 | 1.083089 |
| 3a,7a-Dihydroxycoprostanic acid | 2.04705 | 2.77E-09 | -1.93066 |
| 3-Aminosalicylic acid | 1.28906 | 0.009717 | -1.11357 |
| 3'-AMP | 1.95314 | 0.017969 | -2.83259 |
| 3-Dehydroquinate | 1.6634 | 5.46E-06 | -1.36188 |
| 3-Furoic acid | 1.22993 | 0.021098 | 1.133295 |
| 3-Hydroxy-cis-5-tetradecenoylcarnitine | 1.70995 | 3E-05 | 1.498822 |
| 3-Methylhistamine | 1.46887 | 4.01E-05 | -1.0981 |
| 3-oxoglycyrrhetinic acid | 1.43166 | 0.000592 | 1.158783 |
| 4-(Methylnitrosamino)-1-(3-pyridyl)-1-butanol glucuronide | 2.57851 | 2.38E-06 | 3.288205 |
| 4-Aminohippuric acid | 1.72521 | 1.15E-08 | 1.364965 |
| 4-Hydroxycinnamic acid | 2.05389 | 3.12E-08 | 1.975667 |
| 4-Methoxybenzaldehyde | 1.05098 | 0.012864 | 0.762097 |
| 4-Methylbenzoic acid | 1.43865 | 4.61E-06 | 0.990883 |
| 4-Methylcatechol | 1.45367 | 0.000174 | 1.131202 |
| 4-Methylumbelliferyl acetate | 2.73811 | 1.4E-08 | -3.50924 |
| 4-Trimethylammoniobutanoic acid | 1.21778 | 0.00208 | -0.88824 |
| 5beta-scymnol | 3.29955 | 3.64E-09 | -5.07929 |
| 5-Hydroxyindoleacetic acid | 1.38255 | 0.020644 | -1.40631 |
| 5-Hydroxy-L-tryptophan | 1.4051 | 1.42E-06 | 0.924115 |
| 5-Methoxytryptamine | 1.48854 | 4.86E-05 | 1.133505 |
| 5-Methyldeoxycytidine | 1.45835 | 0.004709 | 1.376606 |
| 5-Methylthioribose | 1.10266 | 0.016851 | -0.88451 |
| 6,8-Diprenylnaringenin | 1.9142 | 2.34E-06 | 1.7942 |
| 6-Acetyl-2,2-dimethyl-2H-1-benzopyran | 1.005 | 0.002003 | 0.560683 |
| 6alpha-Hydroxy-castasterone | 1.4326 | 0.000284 | -1.11629 |
| 6beta-Hydroxytestosterone | 1.95642 | 1.47E-05 | 1.94492 |
| 6-Oxopiperidine-2-carboxylic acid | 1.24585 | 0.006331 | -1.00298 |
| 7-a,25-Dihydroxycholesterol | 1.18271 | 0.000454 | -0.75441 |
| Acebutolol | 1.36253 | 0.000198 | -0.98677 |
| Acetylcholine | 2.16393 | 0.035365 | 3.861497 |
| Ajmalicine | 1.69659 | 0.007258 | 1.893626 |
| Androstenedione | 1.659 | 0.001945 | 1.654082 |
| Androsterone glucuronide | 1.91251 | 6.59E-06 | -1.80814 |
| Aprobarbital | 1.12883 | 0.000118 | 0.625259 |
| Arbekacin | 1.25053 | 0.000704 | -0.87241 |
| Arginyl-Arginine | 1.35843 | 0.003205 | -1.12818 |
| Arginyl-Glutamine | 1.65903 | 3.29E-05 | -1.41089 |
| Ascorbic acid | 1.33033 | 0.014088 | 1.264204 |
| Aspartyl-Leucine | 2.38537 | 0.000788 | -3.31343 |
| Azelaic acid | 1.01216 | 0.004641 | 0.629328 |
| Benzenebutanoic acid | 1.15103 | 0.000997 | 0.742939 |
| Bethanidine | 2.15575 | 0.00051 | -2.65457 |
| Bilirubin | 1.44345 | 0.001476 | -1.24161 |
| Butylparaben | 1.01869 | 0.002038 | 0.591434 |
| Calycanthidine | 2.9399 | 2.36E-12 | 3.938918 |
| Caprylic acid | 1.00856 | 0.001786 | -0.56771 |
| Carisoprodol | 1.14645 | 0.000492 | -0.70528 |
| Casimiroin | 1.93244 | 8.06E-08 | 1.756789 |
| Catechin | 1.08264 | 0.010765 | 0.783996 |
| Chenodeoxycholic acid | 2.916 | 6.91E-08 | -4.03428 |
| Chenodeoxycholic acid glycine conjugate | 2.50831 | 7.91E-06 | -3.1692 |
| Cholesta-4,6-dien-3-one | 2.50617 | 1.41E-11 | -2.86189 |
| Ciclopirox | 1.36135 | 3.06E-05 | 0.921897 |
| cis-4-Hydroxy-D-proline | 1.01334 | 0.027778 | -0.79054 |
| Citric acid | 2.03234 | 0.00012 | -2.22751 |
| Coumesterol | 1.45851 | 2.93E-06 | 1.01558 |
| Cyclohexanecarboxylic acid | 1.95865 | 5.61E-05 | 2.017515 |
| Cyclohexylamine | 1.21208 | 0.004644 | -0.93878 |
| Cyclopentolate | 1.60127 | 0.001031 | -1.48934 |
| D-2-Hydroxyglutaric acid | 1.24406 | 0.004823 | -0.98168 |
| Daidzein | 1.79263 | 0.003351 | 2.006033 |
| D-Aspartic acid | 1.21058 | 0.00537 | -0.94701 |
| Deoxycholic acid | 2.79545 | 2.34E-09 | -3.62883 |
| Deoxypyridinoline | 1.56069 | 0.012411 | 1.731973 |
| D-Galactose | 1.18417 | 0.001084 | -0.79409 |
| Diacetyl | 1.01049 | 0.00203 | -0.578 |
| Diethyl malonate | 2.27219 | 7.28E-05 | 2.745375 |
| Diethyl tartrate | 1.2648 | 4.63E-05 | -0.79382 |
| Digalacturonic acid | 1.22787 | 0.001235 | -0.86649 |
| Dihydrojasmone | 1.2654 | 5.52E-05 | 0.798708 |
| Dihydromethysticin | 1.47524 | 1.25E-05 | 1.070072 |
| Dimethyl sulfoxide | 1.30041 | 0.001397 | 0.992553 |
| Dodecanedioic acid | 1.30457 | 0.000125 | 0.881676 |
| D-Proline | 1.74283 | 1.16E-06 | -1.46312 |
| D-Ribose | 1.75754 | 1.18E-05 | -1.55205 |
| D-Urobilin | 1.604 | 0.010059 | 1.768063 |
| D-Xylitol | 2.05229 | 0.00041 | -2.35644 |
| Ecgonine methyl ester | 1.2316 | 0.002532 | 0.917091 |
| Equol | 1.00356 | 0.009457 | 0.656954 |
| Ethyl acetate | 1.0847 | 0.012549 | -0.81822 |
| Ethyl oleate | 1.20451 | 0.006342 | -0.94991 |
| Ethyl trans-p-methoxycinnamate | 1.61055 | 0.000168 | 1.398745 |
| Eugenol | 1.26685 | 0.001304 | 0.930976 |
| Fenoterol | 1.24707 | 0.000459 | 0.848403 |
| Fludarabine | 1.58902 | 8.23E-07 | -1.19699 |
| Formononetin | 1.78365 | 0.000702 | 1.822149 |
| Fructose 1-phosphate | 2.81747 | 1.89E-05 | -4.08093 |
| Fumaric acid | 1.88387 | 3.05E-06 | -1.74503 |
| Fusarochromanone | 1.27382 | 0.000966 | 0.928917 |
| gamma-Glutamyltyrosine | 1.90763 | 4.71E-05 | 1.884085 |
| Ganciclovir | 1.2249 | 0.003429 | -0.93426 |
| Gentisic acid | 1.21666 | 0.024583 | 1.106731 |
| Gingerol | 1.82876 | 7.52E-06 | 1.669088 |
| Ginkgoic acid | 2.43865 | 4.08E-06 | -2.95003 |
| Glucaric acid | 2.08031 | 0.000444 | -2.45491 |
| Gluconic acid | 1.7926 | 1.92E-05 | -1.63512 |
| Gluconolactone | 1.13354 | 0.011521 | -0.89 |
| Glucosamine | 1.03291 | 0.003175 | -0.63347 |
| Glucose 6-phosphate | 2.27272 | 6.26E-06 | -2.58892 |
| Glutamylaspartic acid | 1.19329 | 0.000261 | -0.74705 |
| Glutamylmethionine | 1.30101 | 0.000472 | -0.92592 |
| Glutamylserine | 1.01622 | 0.048054 | -0.88323 |
| Glutamyltryptophan | 1.51015 | 4.28E-06 | -1.10135 |
| Glutathionylaminopropylcadaverine | 1.68897 | 0.030229 | 2.179326 |
| Glycerol tributanoate | 3.34526 | 0.000748 | -6.51405 |
| Glycocholic acid | 1.6225 | 0.000265 | -1.44677 |
| Glycolic acid | 1.24313 | 0.000497 | -0.84755 |
| Guaiacol | 2.20185 | 1.55E-06 | 2.371031 |
| Hecogenin | 1.36213 | 0.01802 | 1.342816 |
| Hexanal dihexyl acetal | 2.10088 | 3.5E-06 | 2.184348 |
| Hexanoylcarnitine | 2.4103 | 4.87E-07 | -2.80795 |
| Homovanillic acid | 1.32387 | 0.007376 | -1.17394 |
| Humulinone | 1.34537 | 0.000168 | 0.954838 |
| Hydantoin-5-propionic acid | 1.00411 | 0.002485 | -0.57864 |
| Hydrocortisone cypionate | 1.68415 | 3.06E-05 | -1.44557 |
| Indecainide | 1.07061 | 0.01073 | 0.778742 |
| Indole | 1.74457 | 0.013937 | 2.156543 |
| Indole-3-carboxylic acid | 1.93109 | 0.016816 | 2.700416 |
| Indolin-2-one | 1.00444 | 0.036245 | -0.81672 |
| Irisflorentin | 1.07433 | 0.009364 | 0.7743 |
| Isoliquiritigenin | 1.17929 | 0.00067 | 0.762323 |
| Isoniazid pyruvate | 1.32734 | 0.000925 | -1.01227 |
| Isonocardicin C | 1.24382 | 0.00163 | -0.90375 |
| Isovaleric acid | 1.56595 | 1.77E-05 | 1.230957 |
| Jasmonic acid | 1.24171 | 5.07E-05 | 0.763532 |
| L-Acetylcarnitine | 1.09992 | 0.005639 | -0.76351 |
| L-Arogenate | 1.7393 | 2.03E-05 | 1.538978 |
| Levetiracetam | 1.13843 | 0.000507 | 0.694861 |
| Levosimendan | 1.49443 | 0.000104 | -1.17305 |
| L-gamma-glutamyl-L-leucine | 1.02627 | 0.004739 | 0.641811 |
| L-Histidine | 1.51194 | 7.82E-06 | -1.12065 |
| L-Hypoglycin A | 2.21855 | 1.89E-06 | 2.414698 |
| L-Iditol | 1.09137 | 0.010337 | 0.800961 |
| Lindane | 2.06613 | 1.97E-08 | 1.993309 |
| L-Isoleucine | 1.1082 | 0.000771 | -0.66769 |
| Lithocholyltaurine | 1.89108 | 4.67E-05 | -1.85994 |
| L-Malic acid | 1.96675 | 1.15E-06 | -1.87741 |
| Lotaustralin | 1.9831 | 8E-06 | 1.97364 |
| L-Phenylalanine | 1.48783 | 0.003739 | -1.40965 |
| L-Urobilin | 1.20958 | 0.008149 | -0.98253 |
| L-Valine | 1.06653 | 0.036975 | -0.92439 |
| LysoPA(18:1(9Z)/0:0) | 1.3836 | 0.000764 | -1.09069 |
| LysoPC(22:1(13Z)) | 1.05797 | 0.003026 | 0.670573 |
| LysoPC(P-18:0) | 1.36826 | 0.002337 | 1.125848 |
| Lysyl-Leucine | 1.07119 | 0.02114 | -0.83154 |
| Malic acid | 2.59452 | 6.36E-07 | -3.268 |
| Malonate | 1.06936 | 0.005176 | -0.71045 |
| Mammea A/BA | 1.68764 | 8.65E-05 | 1.50621 |
| Mequitazine | 1.56169 | 5.11E-05 | 1.259089 |
| Mesalazine | 1.19613 | 8.48E-05 | -0.71343 |
| Methdilazine | 1.31248 | 0.002639 | 1.059349 |
| Methohexital | 1.93474 | 0.000105 | 2.006207 |
| Mexiletine | 1.28513 | 0.000789 | 0.927018 |
| Midodrine | 1.12956 | 0.007131 | -0.83701 |
| Morphine | 1.30279 | 0.001068 | 0.979624 |
| Moxonidine | 1.57859 | 0.004226 | 1.592112 |
| Myxochelin B | 1.50636 | 6.75E-07 | 1.061171 |
| N6-beta-Aspartyllysine | 1.16328 | 0.000785 | 0.749698 |
| N6-Methyladenosine | 1.56021 | 0.022664 | -1.86666 |
| N-Acetylglutamic acid | 1.49748 | 7.14E-06 | -1.09493 |
| N-Acetylneuraminic acid | 1.17297 | 0.001125 | 0.781575 |
| Nafcillin | 1.48027 | 0.001859 | 1.327931 |
| Nandrolone decanoate | 1.14517 | 0.001066 | -0.72432 |
| Naphazoline | 1.39734 | 0.000356 | 1.070809 |
| Narceine | 1.11351 | 0.048956 | -1.05782 |
| Naringenin | 1.75351 | 0.003938 | 1.940182 |
| Neodunol | 1.02084 | 0.004906 | 0.647736 |
| Neotame | 1.65158 | 1.8E-06 | -1.31548 |
| neriifolin | 1.54969 | 1.9E-05 | -1.19864 |
| N-Ethyl trans-2-cis-6-nonadienamide | 1.35677 | 0.000619 | 1.034935 |
| Niacinamide | 1.42877 | 0.002275 | -1.24404 |
| Nifedipine | 1.01052 | 0.003036 | -0.5891 |
| Nigakinone | 2.42951 | 0.000106 | 3.161917 |
| Nitrendipine | 1.34971 | 0.000235 | -0.96629 |
| Nornicotine | 2.24205 | 2.76E-06 | 2.476813 |
| Northienamycin | 2.23253 | 5.34E-05 | -2.6268 |
| N-Undecanoylglycine | 1.27551 | 0.00503 | -1.04396 |
| Oleandomycin | 2.22146 | 4.42E-08 | 2.325118 |
| Ononin | 1.02086 | 0.000608 | 0.535476 |
| O-Phospho-4-hydroxy-L-threonine | 1.93627 | 0.002156 | 2.283352 |
| O-Phosphoethanolamine | 1.84253 | 0.000376 | -1.90434 |
| Orotic acid | 1.65687 | 0.001796 | -1.6436 |
| Osajin | 1.13324 | 0.000787 | 0.70636 |
| Oxaprozin | 1.06736 | 0.011871 | -0.79019 |
| Oxoglutaric acid | 1.2688 | 0.001032 | -0.92208 |
| Oxymetazoline | 1.41949 | 3.17E-06 | -0.95774 |
| Palmitoylethanolamide | 1.88867 | 0.000654 | 2.025778 |
| p-Aminobenzoic acid | 1.28406 | 9.14E-05 | 0.838873 |
| PC(14:0/14:0) | 2.34366 | 2.43E-07 | -2.63179 |
| PC(18:1(9Z)/18:2(9Z,12Z)) | 1.94063 | 0.001141 | -2.23993 |
| p-Cresol | 1.76846 | 4.12E-07 | 1.486366 |
| Penciclovir | 1.19453 | 7.55E-05 | -0.70613 |
| Pentadecanoic acid | 1.2086 | 0.000917 | -0.82613 |
| Perindopril | 1.55154 | 4.27E-05 | 1.236265 |
| Phenyl acetate | 1.20207 | 3.23E-05 | 0.692972 |
| Phenylglyoxylic acid | 1.29044 | 0.01014 | -1.14669 |
| Phloretin | 1.28729 | 0.002204 | -0.99539 |
| Phosphoric acid | 2.80053 | 1.44E-06 | -3.8465 |
| Phthalic acid | 2.09877 | 1.01E-08 | -2.04898 |
| Pilocarpine | 1.25809 | 0.000361 | 0.851051 |
| Pipecolic acid | 1.87451 | 0.000726 | 2.005667 |
| Pirbuterol | 1.1889 | 0.002087 | 0.843102 |
| Procainamide | 1.36447 | 0.022249 | 1.397885 |
| Propantheline | 1.55353 | 0.000255 | 1.302403 |
| Protoporphyrin IX | 1.59332 | 0.000871 | -1.46956 |
| Pyridoxamine | 1.42958 | 7.85E-06 | 0.993622 |
| Pyrophosphate | 2.99351 | 3.43E-07 | -4.32167 |
| Pyrrolidonecarboxylic acid | 1.02603 | 0.003652 | -0.63335 |
| Quillaic acid | 2.32385 | 4.01E-11 | -2.46106 |
| Quinaldic acid | 1.21493 | 0.012109 | 1.014987 |
| Raffinose | 1.32867 | 0.000145 | -0.92173 |
| Remifentanil | 1.60271 | 3.75E-05 | 1.31796 |
| Retinol acetate | 2.18175 | 2.3E-05 | -2.43875 |
| Rhizocticin B | 3.93524 | 3.73E-11 | -7.1187 |
| Riboflavin reduced | 1.47283 | 0.005761 | 1.390775 |
| Ricinoleic acid | 1.53285 | 0.000171 | 1.258358 |
| Risperidone | 1.8477 | 2.44E-06 | 1.661961 |
| Roxatidine acetate | 1.0968 | 0.008417 | -0.78867 |
| Saccharin | 2.28782 | 2.97E-09 | 2.422697 |
| Scolymoside | 1.07368 | 0.002613 | 0.663631 |
| Shikimic acid | 1.00385 | 0.024051 | -0.74138 |
| Sorbitan laurate | 1.22034 | 0.001338 | -0.85612 |
| Stearic acid | 2.79486 | 3.22E-06 | 3.879452 |
| Succinic acid | 1.78452 | 1.64E-05 | -1.61331 |
| Succinylacetone | 1.39551 | 0.002509 | -1.1965 |
| Taurolithocholic acid 3-sulfate | 1.6748 | 0.001225 | -1.6476 |
| Temozolomide | 1.02258 | 0.001467 | 0.566339 |
| Tetradecanedioic acid | 2.0602 | 8.78E-08 | 2.003539 |
| Thiosulfate | 1.58666 | 0.000497 | -1.4075 |
| Tiglylglycine | 1.87292 | 2.35E-08 | -1.63026 |
| Toluene | 1.37683 | 4.67E-05 | 0.951514 |
| Trandolapril | 2.8749 | 1.46E-07 | -3.95385 |
| trans-Ferulic acid | 1.37656 | 0.003653 | 1.197527 |
| Triclosan | 1.29719 | 0.003511 | 1.057075 |
| Tridemorph | 1.37817 | 0.007922 | -1.28906 |
| Trimetaphosphoric acid | 2.93973 | 3.77E-07 | -4.17486 |
| Trimethaphan | 3.19131 | 0.000297 | -5.69153 |
| Tropinone | 2.07313 | 2.81E-07 | -2.05092 |
| Tryptophanol | 1.24825 | 3.52E-05 | 0.761016 |
| Tryptophyl-Phenylalanine | 1.09989 | 0.002924 | -0.7237 |
| Tuftsin | 1.0041 | 0.039888 | 0.832256 |
| Tyramine | 2.05655 | 7.39E-06 | 2.108725 |
| Ubiquinone-2 | 1.17087 | 0.000495 | 0.735427 |
| Uric acid | 1.57165 | 0.009618 | 1.687339 |
| Valaciclovir | 1.27245 | 0.024935 | -1.21166 |
| Voriconazole | 1.41181 | 0.000101 | 1.040179 |
| Xanthosine | 1.51559 | 0.002445 | -1.37875 |
| Ziprasidone | 1.14735 | 8.69E-05 | 0.644345 |
